# The Effects of Dietary Insect Meal from *Hermetia illucens* Prepupae on Autochthonous Gut Microbiota of Rainbow Trout (*Oncorhynchus mykiss*)

**DOI:** 10.3390/ani9040143

**Published:** 2019-04-02

**Authors:** Simona Rimoldi, Elisabetta Gini, Federica Iannini, Laura Gasco, Genciana Terova

**Affiliations:** 1 Department of Biotechnology and Life Sciences, University of Insubria, Via J.H. Dunant 3, 21100 Varese, Italy; simona.rimoldi@uninsubria.it (S.R.); elisabetta.gini@uninsubria.it (E.G.); fiannini@uninsubria.it (F.I.); 2 Department of Agricultural, Forest and Food Sciences, University of Turin, Largo P. Braccini 2, 10095 Grugliasco, Torino, Italy; laura.gasco@unito.it

**Keywords:** aquaculture, insect meal, black soldier fly, gut microbiome, high throughput sequencing

## Abstract

**Simple Summary:**

The composition of fish-gut microbial communities has been demonstrated to adapt when the host is fed different ingredients. Fishmeal and soy are the conventional protein sources used in aquafeeds. However, these feed options are not sustainable anymore due to the progressive depletion of wild marine fish stocks and the considerable environmental cost of protein-rich terrestrial plant cultivation. In this perspective, insects could be a promising source of protein and may help aquaculture to cope with the increasing global demand for new protein sources, representing the idea of “waste into feed” bioconversion. In this perspective, we evaluated the effects of dietary insect meal from *Hermetia illucens* (Hi) larvae on autochthonous gut microbiota of rainbow trout (*Oncorhynchus mykiss*). Hi larvae were grown on leftover fruit and vegetables provided by a local wholesale market. Three diets, with increasing levels of insect meal inclusion (10%, 20%, and 30%) and a control diet without insect meal were tested in a 3-month fish feeding trial. The data showed that feeding insects influences the intestinal bacterial communities, thus improving fish gut health. In our opinion, these findings represent a precious tool for future research on salmonid’s microbial communities and their interaction with diet and the host.

**Abstract:**

This study evaluated the effects of dietary insect meal from *Hermetia illucens* larvae on autochthonous gut microbiota of rainbow trout (*Oncorhynchus mykiss*). Three diets, with increasing levels of insect meal inclusion (10%, 20%, and 30%) and a control diet without insect meal were tested in a 12-week feeding trial. To analyze the resident intestinal microbial communities, the Illumina MiSeq platform for sequencing of 16S rRNA gene and QIIME pipeline were used. The number of reads taxonomically classified according to the Greengenes database was 1,514,155. Seventy-four Operational Taxonomic Units (OTUs) at 97% identity were identified. The core of adhered intestinal microbiota, i.e., OTUs present in at least 80% of mucosal samples and shared regardless of the diet, was constituted by three OTUs assigned to *Propiobacterinae*, *Shewanella*, and *Mycoplasma* genera, respectively. Fish fed the insect-based diets showed higher bacterial diversity with a reduction in *Proteobacteria* in comparison to fish fed the fishmeal diet. Insect-meal inclusion in the diet increased the gut abundance of *Mycoplasma*, which was attributed the ability to produce lactic and acetic acid as final products of its fermentation. We believe that the observed variations on the autochthonous intestinal microbiota composition of trout are principally due to the prebiotic properties of fermentable chitin.

## 1. Introduction

The global population will reach 9 billion by 2050 with a consequent inevitable increase of food demand, especially for animal-based protein sources [1]. In this scenario, the main challenge of the world food-producing sector will be to provide an affordable, safe, and sustainable food supply. To achieve this goal, a sustainable food production based on low human-interest alternative feed ingredients that require less land, water, and energy resources is necessary. Particularly, aquaculture is the fastest growing food-producing sector in the world, with a worldwide finfish production increased by 90% during the last decade (2004–2014) [2]. Fishmeal (FM) and soy are the conventional protein sources used in aquafeeds. However, these feed options cannot be anymore sustained due to the progressive depletion of wild marine fish stocks [2,3,4,5] and to the considerable environmental cost of protein-rich plant cultivation [6,7].

All this has encouraged the search for more sustainable protein sources characterized, at the same time, by good nutritional values for cultured fish, above all for carnivorous species, which have high protein requirements (40–45% for trout and marine finfish). The most promising animal alternatives to FM are nonruminant processed animal proteins [8,9,10] and insect meals [11,12,13,14]. In recent years, the attention of the aquafeed industry has mainly addressed the use of insects, since compared to other animal protein sources, they—in particular, flies—show several advantages. Besides being a natural part of the diet for wild fish, insects easily grow on organic waste substrates showing a high feed conversion efficiency. They have low ecologic impact and greenhouse gases and ammonia production, low risk of transmitting zoonotic infections, and relatively low interest from human consumers [11,12,15,16]. Additionally, insects have good nutritional value, since they contain high level of proteins (60–80%) and lipids (31–43%) and are rich in essential amino acids (EAA), vitamins, and minerals [12]. Recently, seven insects (two flies, two mealworms, and three cricket species) have been authorized as fish feed by EU commission regulation (2017/893-24/05/2017). Of these, black soldier fly, *Hermetia illucens* (Hi), is one of the most promising species. Hi larvae grow on different organic substrates, consuming twice their weight each day, and, at prepupae stage, they contain a very high percentage of protein (36–48% DM) and fat (31–33% DM), and their essential amino acid profile is very similar to that of FM [12]. The potential of *H. illucens* as a valuable feed ingredient has been established for several commercially important freshwater and marine fish species [17,18,19,20,21,22,23,24]. These studies have demonstrated that *H. illucens* prepupae meal can replace up to 40% of FM in salmon (*Salmo salar*) and sea bass (*Dicentrarchus labrax*) [18,19] and up to 50% in rainbow trout (*Oncorhynchus mykiss*) [24] without compromising fish growth performances.

However, although several studies regarding the effect of dietary insect meal on fish growth are available in the literature, less is known on its impact on the gut microbiota of fish. Like in mammals, the intestinal microbiota of fish plays important role in host metabolism, nutrition, immunity, and health of fish [25,26]. It has been largely demonstrated that substitution of FM with alternative protein sources, such as plants, yeast, and animal byproducts, alters the diversity and abundance of intestinal bacteria in salmonids [9,10,27,28,29,30,31].

A positive modulation of fish intestinal microbiota by dietary inclusion of insect meal is reasonably expected for at least three reasons. First, insects are rich in chitin a mucopolysaccharide polymer structurally analogous to cellulose [32]. Generally, chitin is not digestible by fish and may act as an insoluble fiber with potential prebiotic properties. Chitin can be fermented by healthy gut microflora, thus contributing to the synthesis of vitamins and other metabolites such as short-chain fatty acids (SCFAs), the main energy source of colonocytes [33]. Secondly, chitin has an antimicrobial and bacteriostatic activity against several pathogenic Gram-negative bacteria species, such as *Escherichia coli* and *Anaerorhabdus furcosa* [34,35,36]. Thirdly, black soldier prepupae are particularly rich in lauric acid (C12:0), a medium-chain fatty acid (MCFA) with antimicrobial properties versus Gram-positive bacteria [37,38,39]. Actually, the latest studies seem to support this hypothesis. Feeding with black soldier larvae meal increases diversity and alters the composition of gut bacteria in rainbow trout [14,24,40]. Compared to FM control, insect-fed groups resulted in higher abundance of phyla *Firmicutes* and *Actinobacteria* with lower abundance of *Proteobacteria* [14,40].

However, excepting the above-mentioned studies, research on *H. illucens* meal impact on fish gut microbiota remains scarce. Accordingly, the present research aimed to investigate the effects of *H. illucens* meal-based diets on autochthonous (adhered) gut microbiota of rainbow trout. Data relating to allochthonous (transient) intestinal bacteria are not included in this study, but they were recently reported by Terova et al. [14]. For the feeding trial, defatted insect meal from *H. illucens* prepupal larvae grown on a substrate of fruit and vegetables was used. The high-throughput sequencing analysis of 16S rRNA gene was applied to characterize the resident intestinal microbial communities of rainbow trout fed for 12 weeks with four different diets: three experimental formulations with increasing amounts of *H. illucens* meal (Hi 10%, Hi 20%, and Hi 30%) and one control FM-based feed (Hi 0%).

## 2. Materials and Methods

### 2.1. Ethics Statement

All procedures involving fish comply with the guidelines of the European Union Council (2010/63/EU) for the use of experimental animals and have been approved by the Italian Ministry of Health [REF:1190/2016PR (response of Prot. Nr. 344C6.5 of 13/10/2016) in accordance to the Art.31 of D.lgs.26/2014)].

### 2.2. Experimental Diets

A partially defatted *Hermetia illucens* (Hi) larvae meal was used to formulate four isonitrogenous (crude protein (CP): about 49 g/100 g dry matter), isolipidic (ether extract (EE): about 18 g/100 g dry matter), and isoenergetic (gross energy about 19.87 MJ/kg DM) diets based on the control diet containing 60% fishmeal (Hi 0). Specifically, the three experimental diets contained 10% (Hi 10), 20% (Hi 20), and 30% (Hi 30) of Hi meal in partial replacement of fishmeal. All feeds were prepared through cold pelleting at the experimental facility of the Department of Agricultural, Forest and Food Science (DISAFA) of the University of Turin (Torino, Italy). Details concerning feed manufacturing have been described by Terova et al. [14]. However, briefly, all grounded ingredients were mixed with oils; water was then added to the mixture to attain an appropriate consistency for pelleting. Each diet was cold pelleted using a 2.5 mm die meat grinder. After pelleting, the diets were dried at 50 °C for 48 h and then stored in dark bags at −20 °C until utilisation. The ingredients and proximate composition of diets are reported in Table 1. Due to differences in chemical composition between Hi and fishmeal and to maintain isonitrogenous, isolipidic, and isoenergetic diets, the level of inclusion of some other dietary ingredients such as fish oil and wheat bran were modified with the increase of HI inclusion in the diets.

### 2.3. Feeding Trial and Sampling

The detailed description of experimental trial can be found at Terova et al. [14]. However, briefly, a total of 348 rainbow trout (*Oncorhynchus mykiss*) with an initial mean body weight of 66.5 ± 1.7 g were distributed in 12 indoor rectangular fiber-glass tanks and fed for 12 weeks with four experimental diets in triplicate (three tanks/diet). Feed was manually distributed and the feeding rate was restricted to 1.5% of biomass for the entire duration of the experiment.

At the end of the trial, three fish per replicate (nine fish/diet) were euthanized with 320 mg/L of tricaine-methasulfonate (MS-222, Sigma-Aldrich, Milan, Italy). Before dissection, the external surface of each fish was wiped with 70% ethanol to avoid any accidental contamination from external body surface microflora. The intestine (excluding pyloric ceca) was aseptically removed with alcohol-disinfected instruments from each fish and the fecal matter removed by squeezing. The autochthonous (adhered) intestinal bacteria were then collected by scraping intestinal mucosa with a sterile cotton swab. The tip of the swab was immediately transferred in a sterile Eppendorf tube containing 200 μL of XpeditionTM Lysis/Stabilization Solution (Zymo Research, Irvine, CA, USA) and vortexed several times to facilitate the bacteria releasing in the solution. After 2 h, the swab was removed, and the solution stored at room temperature until DNA extraction.

### 2.4. Bacterial DNA Extraction

DNA was extracted from 200 μL of bacterial suspension using DNeasy PowerSoil^®^ Kit (Qiagen, Milan, Italy), according to the manufacturer’s instructions. The samples were lysed in PowerBead Tube by means of a TissueLyser II (Qiagen) for 2 min at 25 Hz. As the negative control of the extraction procedure, a sample with only lysis buffer was processed in parallel with samples. The concentration of extracted DNA was measured using NanoDrop^TM^ 2000 Spectrophotometer (Thermo Scientific, Milan, Italy) and stored at −20 °C until the PCR reaction was performed.

### 2.5. Illumina 16S Metagenomic Sequencing Library Construction

16S ribosomal RNA gene amplicon libraries were prepared using a primer pair sequences for the V3–V4 region following the Illumina protocol “16S Metagenomic Sequencing Library Preparation for Illumina MiSeq System” (#15044223 rev. B). Bacterial 16S rRNA gene amplicons were generated from 50 ng of microbial genomic DNA in 25 μL PCR using Platinum^®^ Taq DNA Polymerase High Fidelity kit (Thermo Fisher Scientific, Italy) and tailed forward and reverse primer Pro341F (5′-CCTACGGGNBGCASCAG -3′) and Pro805R (5′-GACTACNVGGGTATCTAATCC -3′) selected by Takahashi et al. [41]. The expected size on Agilent 2100 Bioanalyzer trace after the amplicon PCR step was ~550 bp. The entire procedure for 16S rRNA gene library preparation and sequencing is described in Rimoldi et al. [10]. Briefly, Illumina paired-end adapters with unique Nextera XT indexes were ligated to 16S amplicons using Nextera XT Index Kit (Illumina, San Diego, CA, USA). All libraries were then subjected to quality control using qPCR using KAPA Library Quantification Kits Illumina^®^ Platforms (Kapa Biosystems Ltd., London, UK), pooled at equimolar concentrations, and diluted to 6 picomolar. Pooled libraries were then multiplexed and sequenced on an Illumina MiSeq platform (Illumina, San Diego, CA, USA) with paired-end 2 × 300 bp sequencing chemistry.

### 2.6. Metagenome Data Analysis

Raw FASTQ sequencing data were processed using the open-source bioinformatics pipeline Usearch 9.2 (Edgar, 2010) [42] at the default setting. To reconstruct the original amplicons, overlapping R1 and R2 paired reads were merged using the ‘fastq_mergepairs’ command and filtered for base quality (Q > 30) by means of the ‘fastq_filter’ command. The ‘fastx_uniques’ denoising command was used on remaining high-quality reads and denoised sequences with 97% or higher identity were de novo clustered into Operational Taxonomic Units (OTUs). Chimeric sequences were removed using ‘cluster_otus’ script with UPARSE-OUT algorithm. Taxonomy was assigned to each OTU using ‘usearch_global’ script against the Ribosomal Database Project (RDP) (http://rdp.cme.msu.edu). Only the OTUs that represented at least 0.005% of total reads were kept. The taxonomical classification was performed down to the genus level. OTUs assigned to chloroplasts and mitochondria were removed from the downstream analysis since of eukaryotic origin. Alpha and beta diversity statistics have been performed using Usearch script ‘alpha_div’ and ‘beta_div’, respectively.

Alpha diversity was calculated based on a rarefied OTU table (rarefied at the lowest sample size) using diversity metrics ‘richness’, ‘Chao1’ and ‘Shannon’, and evenness metrics ‘Simpson’ and ‘berger_parker’. The distances among bacterial communities (beta diversity) based on both OTUs presence and their abundances or on presence and absence alone were represented using a Bray–Curtis and binary Bray–Curtis matrix, respectively. The dissimilarity matrices were visualized by Non-Metric Multidimensional Scaling (NMDS) plots.

The common core microbiome (OTUs shared regardless of the diet and found in at least seven out of the nine samples per dietary group) was identified and visualized by a Venn diagram drawn using the web tool http://bioinformatics.psb.ugent.be/webtools/Venn/.

### 2.7. Statistical Analysis

All data were first checked for normality and homoscedasticity by Shapiro–Wilk’s and Levene’s test, respectively. Depending if normality of the data was satisfied or not, differences between groups were analysed by one-way ANOVA followed by Tukey–Kramer post-hoc test or by nonparametric Kruskal–Wallis and Mann–Whitney test with Bonferroni correction for multiple testing. Statistical significance was set at *p* < 0.05. All the statistical analyses were performed using Past3 software (Hammer et al. 2001) [43].

The number of reads across samples was normalized by sample size and the relative abundance (%) of each taxon was calculated. Only those taxa with an overall abundance of more than 1% (up to order level) and 0.01% at family and genus level were considered for statistical analysis. Before being statistically analyzed, the resulting microbial profiles were calculated as the angular transformation (arcsine of the square root).

Multivariate analysis of beta diversity was tested using Similarity Percentage Analysis (SIMPER) followed by one-way Permutational Multivariate Analysis of Variance (PERMANOVA) and Analysis of Similarities (ANOSIM) using Bray–Curtis index at 999 permutations with diet as factor. Bonferroni correction was applied to determine significant differences (*p* < 0.05) in gut microbial communities between diets.

## 3. Results

### 3.1. Structure of Autochthonous Intestinal Bacterial Communities

**Figure 1:**
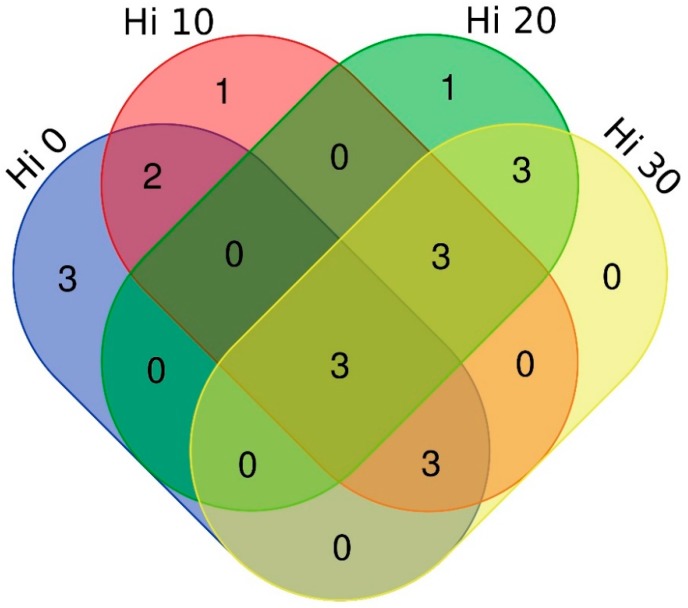
Venn diagram representing unique and shared Operational Taxonomic Units (OTUs) among all dietary groups. The core microbiome was defined as the OTUs present in 80% of the samples regardless of diet.

The 36 intestinal mucosa samples from rainbow trout fed the FM and Hi meal diets were subjected to Illumina Miseq sequencing of the V3–V4 region of the 16S rRNA gene. After data quality filtering, the total number of sequences taxonomically classified was 1,514,155, which corresponded to 42,060 ± 12,839 (mean ± SD) reads per fish. A total of 74 OTUs at 97% identity were identified in trout mucosa samples (Table S1). All sequencing data, as fastq files, were deposited in the European Nucleotide Archive (EBI ENA) public database as accession project code PRJEB28677 and sample accession codes from ERS3037754 to ERS30378369). The core of adhered intestinal microbiota, i.e., OTUs present in at least 80% of mucosal samples and shared regardless of the diet, was constituted by only 3 OTUs assigned to *Propiobacterinae*, *Shewanella* and *Mycoplasma* genera, respectively (Figure 1).

**Figure 2:**
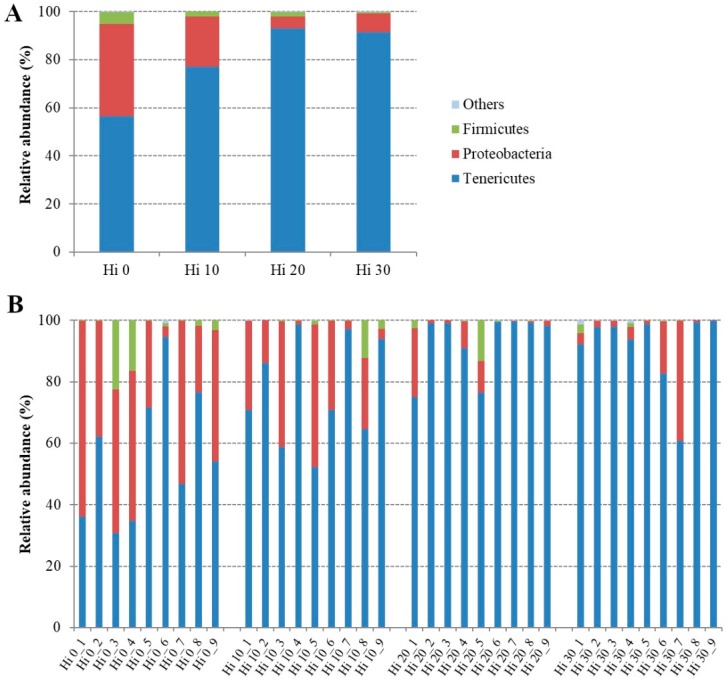
Relative abundance (%) of the overall most prevalent bacterial phyla in each dietary groups (**A**) and individual fish (**B**). In the figure, all taxa with an overall abundance of ≥ 1% were reported.

**Figure 3:**
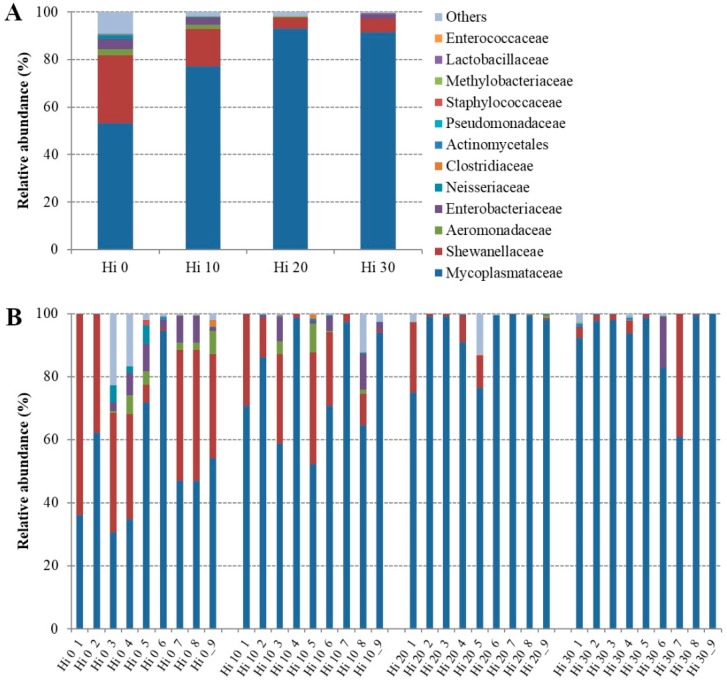
Relative abundance (%) of the overall most prevalent bacterial families in each dietary groups (**A**) and individual fish (**B**). In the figure, all taxa with an overall abundance of ≥ 0.01% were reported.

**Figure 4:**
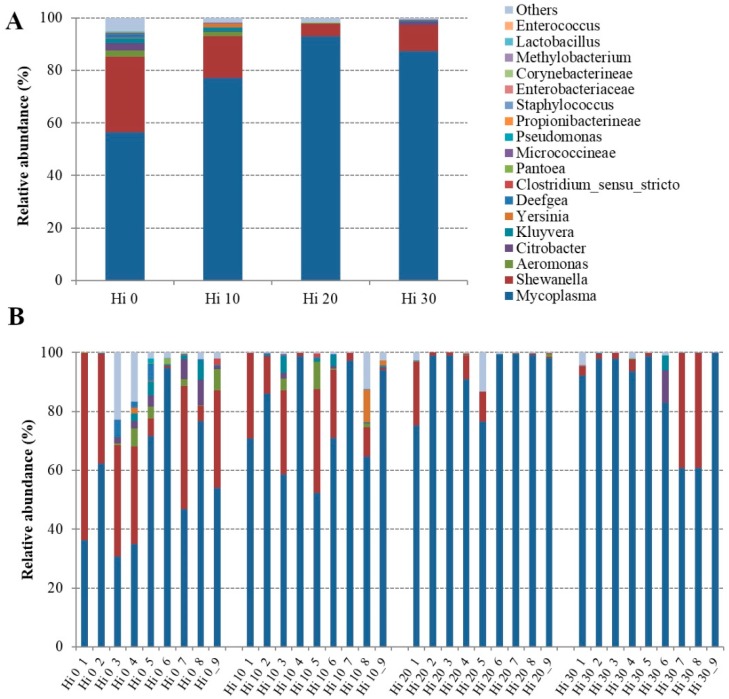
Relative abundance (%) of the overall most prevalent bacterial genera in each dietary groups (**A**) and individual fish (**B**). In the figure, all taxa with an overall abundance of ≥ 0.01% were reported.

The bacterial OTUs found in fish mucosa samples were mainly comprised of six phyla, seven classes, ten orders, 15 families, and 22 genera (Table S1). However, considering only the most representative taxa, the overall autochthonous intestinal microbial community consisted of three phyla, four classes, four orders, 12 families, and 22 genera. The microbial profiles of each dietary group and individual fish are presented at the phylum (Figure 2), family (Figure 3), and genus (Figure 4) level.

To calculate alpha rarefaction indices, a sequencing depth of 17,800 reads per sample was considered. Analysis of alpha-diversity of gut bacteria showed that entropy, indicated by Shannon evenness (Shannon_e) and Simpson indices, significantly increased (*p* < 0.05) for fish fed insect diets Hi 20 and Hi 30, but not Hi 10, in comparison to the control Hi 0 group (Table 2). Similarly, Hi 20 and Hi 30 samples showed the highest values for reciprocal Berger_parker index (1/d). An increase of this index value corresponds to an increase in bacteria diversity and a decrease in dominance. Conversely, Hi meal administration did not significantly affect either the number of observed OTUs or species richness (Chao1 index) (Table 2).

Insect diets had an overall effect on the beta-diversity and composition of gut mucosa-associated bacteria both in presence/absence (binary Bray–Curtis matrix) (PERMANOVA: F = 6.785, *p* = 0.001; ANOSIM: R = 0.4805, *p* = 0.001) and relative abundance (Bray–Curtis matrix) (PERMANOVA: F = 5.202, *p* = 0.001; ANOSIM: R = 0.2522, *p* = 0.002) of OTUs (Table 3). SIMPER analysis showed that, compared with control Hi 0, fish fed Hi 20 diet were the most dissimilar followed by fish fed Hi 30 and Hi 10 diets (Table 3). Although the gut microbial communities were significantly influenced by diet, the found percentages of dissimilarity between control and Hi diets were relatively low. They ranged, indeed, from 32.7 to 26.1% and from 19.2 to 16.8 %, depending on if OTUs abundance was or not taken into account, respectively. The results of pairwise comparisons are summarized in Table 3.

**Figure 5:**
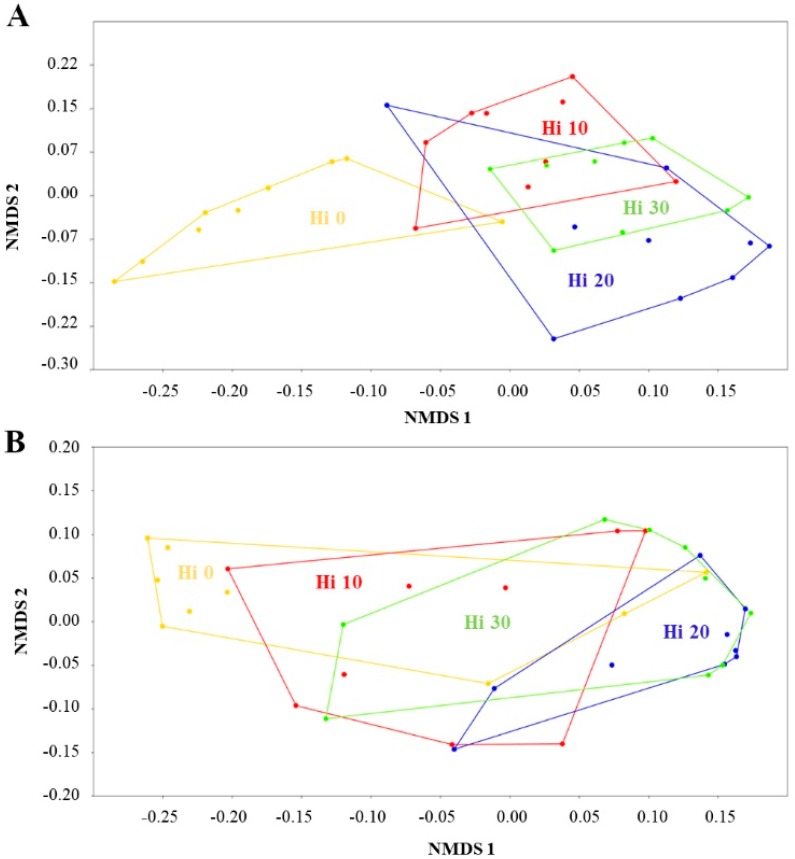
Non-metric multidimensional scaling (NMDS) with 2D Binary Bray–Curtis (**A**) and Bray–Curtis (**B**) index of bacterial OTUs found in intestinal mucosa samples of rainbow trout fed fishmeal (Hi 0) or *H. illucens* larvae meal (Hi 10, Hi 20, Hi 30) diets.

The NMDS plots of binary Bray–Curtis (Figure 5A) and Bray–Curtis (Figure 5B) dissimilarity data agreed with permutational multivariate analysis showing slight clustering in relation to diet. Fish fed Hi 0 diet grouped separately from fish fed Hi 20 (Figure 5A,B). Interestingly, fish fed Hi meal, regardless of dietary inclusion level, formed a separate cluster from the control due to the presence/absence of specific OTUs (Figure 5A).

### 3.2. Dietary Modulation of Autochthonous Intestinal Microbiota

The mucosa-adhered microbial community of our trout was mainly dominated, regardless of the diet, by three phyla: *Tenericutes*, *Proteobacteria*, and *Firmicutes* (Figure 2, Table 4). Among them, *Tenericutes* were the most abundant bacteria in all samples with a relative abundance ranged between 91 and 56%, followed by *Proteobacteria* (38–5%), and *Firmicutes* (0.5–5%). At the phylum level, the amounts of *Tenericutes* and *Proteobacteria* were significantly influenced (*p* < 0.05) by insect meal inclusion in the diet. Specifically, compared to control diet, *Tenericutes*, essentially assigned to *Mollicutes*, significantly increased in fish fed Hi 20 and Hi 30 diets, on the contrary, in the same groups, the amount of *Proteobacteria*, mainly represented by *Gammaproteobacteria* class, diminished (Table 4). At the family and genus level, statistically significant differences in relative abundance of bacterial OTUs were principally found between Hi 0 and Hi 20 dietary groups. Fish fed the Hi 20 diet had a significantly lower percentage of bacteria belonging to *Shewanellaceae*, *Enterobacteriaceae*, and *Neisseriaceae* than fish of the control group (Figure 3, Table 4).

Relative abundance of *Aeromonadaceae* decreased for both Hi 20 and Hi 30 diets, whereas the *Mycoplasmataceae* amount increased in these samples (Table 4). Accordingly, fish fed diet Hi 20 showed a significant enrichment in bacteria assigned to *Mycoplasma* genus (Figure 4, Table 4). Conversely, the relative abundance of *Shewanella*, *Citrobacter*, *Kluyvera*, and *Deefgea* genera was significantly reduced in resident intestinal microflora of trout fed Hi 20 diet in comparison to the FM dietary group. Lastly, only dietary inclusion of 30% of Hi meal caused a significant reduction of bacteria belonging to *Aeromonas* genus in the intestine of our trout (Table 4).

## 4. Discussion

Insect proteins represent a more sustainable alternative than plants to fishmeal in aquafeeds due to their low environmental footprint [44]. Their value as ingredients in fish feed has been widely reviewed, demonstrating no negative effect on fish growth performances when used in place of fish meal in the diet [12,13,45,46,47]. However, research on insect meal impact on gut microbiota of fish is still limited. Therefore, the present study could contribute to fill this gap of knowledge by investigating for the first time the effects of feeding *Hermetia illucens* larvae meal on the mucosa-adhered microbiota of rainbow trout. The mucosa-associated bacterial community, indeed, is expected to have greater impact on host metabolism and health status than transient intestinal bacteria. Bruni et al. [24] firstly explored the dietary Hi meal’s effects on autochthonous microbiota of trout. However, in that study, the gradient gel electrophoresis (DGGE) technique was used which can detect lower number of bacterial species than the high throughput sequencing (Illumina MiSeq) used in the present research. Indeed, studies investigating fish gut microbiota differ to each other at many levels, including methods of microbiome analysis.

In the present study, nine individual fish per diet were used to evaluate autochthonous gut bacteria by high-throughput sequencing. Four diets characterized by an increasing percentage of *H. illucens* larvae meal (Hi 0, Hi 10, Hi 20; Hi 30) in replacement of fishmeal, were tested.

Results obtained from our metagenomic analysis indicated that the most abundant phylum in rainbow trout, regardless of the diet, was *Tenericutes*. Specifically, within this phylum, the *Mollicutes* were the dominant class that, in turn, was exclusively represented by the *Mycoplasma* genus. Recent studies have reported that *Tenericutes* are the prominent phylum, with *Mycoplasma* being the dominant genus, in the distal intestine of rainbow trout, suggesting that trout could be a specific host for this microbe [48,49]. *Mycoplasma* is usually difficult to isolate by conventional microbiological culture methods but using NGS approaches, it has been possible to reveal *mycoplasma* dominance in the intestine of trout. Furthermore, our sequencing results confirmed the results of previous studies, i.e., bacterial communities adhering to mucosa differ from the transient (allochthonous) microflora in fish intestine [24,28,50,51]. Indeed, in a very recent paper of our group [14], *Proteobacteria*, *Firmicutes*, and *Actinobacteria*, in this descending order of abundance, mainly dominated the allochthonous gut microbial community of trout. Interestingly, likewise Bruni et al. [24] OTUs attributable to lactic acid bacteria (*Firmicutes* phylum), were only found in high amount in the gut content samples of trout fed insect meal [14], but were practically absent in gut mucosa of the same fish. Conversely, in line with previous studies in rainbow trout [24,52], a high number of bacteria belonging to *Proteobacteria* phylum (mainly represented by *Gammaproteobacteria*) harboured trout intestinal mucosa. The most abundant *Gammaproteobacteria* detected were associated to the genera *Shewanella*, *Aeromonas*, *Citrobacter*, and *Kluyvera*. The same *Gammaproteobacteria* genera, in addition to *Acinetobacter*, were found by Bruni and colleagues [24] in mucosa-associated microbiota of trout.

Differences between the present and the previously published study of our group [14] are not limited to the most abundant phyla. In mucosa samples, the number of OTUs was significantly lower than in gut digesta samples obtained from the same fish, i.e., 74 OTUs against 450 OTUs [14]. These data are in line with Kim et al. [50], who found in bacteria community hosted by gut mucosa a smaller biodiversity than in the intestinal content. This means that the abundance and diversity of mucosa-adhered bacterial populations could be quite different from the allochthonous microbiota, indicating that some microbial species poorly colonize gut mucosal layer.

Although autochthonous bacterial communities were dominated by the same phyla irrespective of the diet, the present study showed that they are plastic and can be modulated by dietary inclusion of insect meal and perhaps, even partly modified by the other two components that simultaneously decreased in our diets, i.e., soybean oil and wheat bran. This is in agreement with the results of the majority of studies that have assessed alternative dietary protein sources in salmonids [9,10,27,28,31]. In particular, biodiversity parameters (Shannon and Simpson evenness indices) were significantly increased by dietary supply of 20% of insect meal in our study, whereas the same diet did not significantly increase the bacterial richness (chao 1 index). Our findings are in line with previous studies that have evaluated the effects of dietary inclusion of Hi meal in trout [14,24,40]. In these studies, the bacterial biodiversity was positively correlated to insect meal inclusion, too. Likewise, an increase in bacterial alpha diversity was obtained by adding krill or chitin in the diet of salmonids [53,54]. Therefore, by taking into account the chitin content in the insect meal, our results should not be surprising. Chitin is an indigestible mucopolysaccharide polymer, structurally analogous to cellulose and it can act as a prebiotic increasing gut microbiota diversity. High bacterial diversity is generally considered as an indicator of a healthy gut. Reduced diversity, instead, is frequently related to dysbiosis and the risk of diseases, as there is limited bacterial competition for nutrients and colonization by incoming enteric pathogens [55,56].

The NMDS plots of Bray–Curtis and binary Bray–Curtis dissimilarity matrices displayed a slight clustering of fish fed Hi meal from control fish fed without Hi and this was statistically validated by multivariate tests PERMANOVA and ANOSIM. Our data revealed that including Hi meal in the diet caused a significant reduction of mucosa-adhered *Proteobacteria* (phylum of Gram-negative bacteria containing pathogens), predominantly belonging to the *Gammaproteobacteria* class, in comparison to control fish group. Similarly, with respect to the control fish group, feeding insect meal resulted in a lower abundance of *Proteobacteria* in the gut digesta of trout, too [14], and the same result was found in the mixed luminal content and mucosa-adhered trout microbiota in the study of Huyben et al. [40]. Consistently, previous studies in trout reported that the presence of *Proteobacteria* was favoured by an animal protein-based diet, rather than by a vegetable diet rich in fiber [27,31]. Therefore, the *Proteobacteria* decrease in insect meal-fed groups could be due to chitin that is a form of insoluble fiber. Actually, there are several evidences supporting antimicrobial and bacteriostatic properties of chitin and deacetylated chitin derivatives against several harmful Gram-negative bacteria [34]. In Atlantic cod (*Gadus morhua*) and hybrid tilapia, the supply of chitin decreased growth of pathogenic bacterial species, such as *E. coli*, *A. furcosa,* and *A. hydrophila*, respectively [35,36].

In our study, microbiota of trout fed with Hi meal showed a reduction of *Gammaproteobacteria*, mainly represented by genera *Shewanella*, *Aeromonas*, *Citrobacter*, and *Kluyera*. *Shewanella* spp., especially *S. schegeliana*, which has interesting enzymatic activities, being an omega-3 fatty acid-producing bacteria [57,58]. On the other hand, *Aeromonas* and *Citrobacter* genera include potential pathogen species, such as *A. salmonicida*, *A. hydrophyla*, *C. freundii*, and *C. braakii*, that cause diseases in fish. Therefore, the growth inhibiting of potential pathogen genera represents a positive effect of insect meal inclusion in the trout diet.

Interestingly, all genera that were adversely affected by insect-based diets were Gram-negative bacteria. Accordingly, Vogel et al. [59] detected, by standard plate-growth inhibition assay, a strong antimicrobial activity against Gram-negative bacteria of the aqueous extracts of *H. illucens* larvae specifically reared on high-protein (brewer’s grains) and cellulose diets, thus affirming a diet-dependent antimicrobial activity of *H. illucens* extracts against the bacterial species.

Black soldier larvae used in our research were mass-reared on a fruit and vegetable substrate rich in cellulose. This could explain the bactericidal effect on Gram-negative bacterial genera harboured in the intestinal mucosa of trout fed with insect meal. In agreement, Bruni et al. [24] found that OTUs related to *Aeromonas rivipollenis* were only abundant in the control fish group, but unlike us, the insect-fed trout were rich in bacteria related to *Citrobacter*, *Pseudomonas*, and *Delftia* genera. However, their partially divergent results could depend on the limited discriminatory power of the DGGE technique applied.

The increased number of bacteria belonging to *Mycoplasma* genus found in the gut of our fish fed Hi 20 and Hi 30 diets should not have negative consequences on fish health, but rather it could bring benefits. As mentioned, *Mycoplasma* is specifically adapted to the gastrointestinal environment of farmed rainbow trout [47,48]. This genus includes Gram-positive bacteria that are closely related to the *Bacilli*/*Clostridium* branch of the *Firmicutes* phylum and are characterized by extremely small genome (~580 Kbp). Due to their genome size, it is improbable that they could carry out complex metabolic functions within the fish intestine, but perhaps they are obligate commensal microorganisms of the gut ecosystem. It has been reported that *Mycoplasma* bacteria produce lactic acid and acetic acid as their major metabolites [60]. Thus, the dominance of *Mycoplasma* in the intestine of trout could be considered a result of a long-established symbiosis in which this microbe benefits from easy access to a multitude of fermentable substrates and the host benefits from the final metabolites produced by bacterial fermentations. A recent study in trout found a major disease susceptibility associated with decreased *Mycoplasma* levels in the gut [61], whereas in Chinook salmon, the abundance of potentially pathogenic *Vibrio* appeared to be inversely correlated with the presence of *Mycoplasma* [62]. These evidences support the hypothesis that Mycoplasma has a beneficial action on host health by producing antibacterial compounds, such as lactic acid. However, further research on potential functional role of symbiotic bacteria is undoubtedly required.

## 5. Conclusions

In summary, this study showed the effects of dietary *Hermetia illucens* larvae meal on the resident intestinal microbiota of rainbow trout using Illumina high-throughput sequencing. Altogether, our results indicate that feeding insect meal influences the trout intestinal bacterial community, thus improving fish gut health. Fish fed the insect-based diets had higher bacterial diversity, with a reduction in *Proteobacteria* in comparison to fish fed the fishmeal diet. Insect-meal inclusion in the trout diet increased the gut abundance of *Mycoplasma*, which was attributed to the ability to produce lactic and acetic acid as final products of fermentation. We believe that the observed variations on the autochthonous intestinal microbiota composition of trout are mainly due to the prebiotic properties of fermentable chitin. In our opinion, these findings represent a precious tool for future research on salmonid microbial communities and their interactions with diet and the host.

## Figures and Tables

**Table 1:** Diet formulation and proximate composition (modified from Terova et al. [14]).

	*H. illucens* Meal	DIET			
		Hi 0	Hi 10	Hi 20	Hi 30
Ingredients (% as it)					
Fishmeal ^a^		60	54	48	42
*Hermetia illucens* meal		0	10	20	30
Fish oil		7	7	7	7
Soybean oil		5	4	3	2
Wheat bran		10	7	4	1
Wheat meal		4	4	4	4
Starch gelatinized D500		11	11	11	11
Vitamin premix ^b^		1.5	1.5	1.5	1.5
Mineral premix ^c^		1.5	1.5	1.5	1.5
Proximate composition (g/100g DM)					
DM	89.82	89.03	88.62	87.70	86.74
CP	48.62	49.07	49.88	49.48	49.03
EE	20.58	17.73	17.98	17.95	17.56
Ash	8.74	13.71	14.16	14.48	14.26
CHI	4.96	0.00	0.50	0.99	1.51
NFE	17.10	19.48	17.49	17.10	17.64

^a^ Fish meal was purchased from Corpesa S.A. (Santiago, Chile). Proximate composition (% as-fed basis): 91.3 DM; 65.8 CP; 9.4 EE; 15.5 Ash. ^b^ Vitamin premix (IU or mg/kg diet) (Granda Zootecnici Srl, Savigliano, Italy): DL-α tocopherol acetate 60 IU; sodium menadione bisulphate 5 mg; retinyl acetate 15,000 IU; DL-cholecalciferol 3000 IU; thiamin 15 mg; riboflavin 30 mg; pyridoxine 15 mg; vitamin B_12_ 0.05 mg; nicotinic acid 175 mg; folic acid 500 mg; inositol 1000 mg; biotin 2.5 mg; calcium pantothenate 50 mg. ^c^ Mineral premix (g or mg/kg diet) (Granda Zootecnici Srl): bicalcium phosphate 500 g, calcium carbonate 215 g, sodium salt 40 g, potassium chloride 90 g, magnesium chloride 124 g, magnesium carbonate 124 g, iron sulfate 20 g, zinc sulfate 4 g, copper sulfate 3 g, potassium iodide 4 mg, cobalt sulfate 20 mg, manganese sulfate 3 g, sodium fluoride 1 g. DM: Dry matter; CP: Crude protein; EE: Ether extract; CHI: chitin; NFE: Nitrogen-free extract (calculated as 100 − (CP + EE + Ash + CHI).

**Table 2:** Number of reads per sample assigned to OTUs and alpha diversity metrics values (normalized at the lowest sample size: 17,800 sequences) of gut microbial community of rainbow trout fed Hi 0 (*n* = 9), Hi 10 (*n* = 9), Hi 20 (*n* = 9), and Hi 30 (*n* = 9) diets for 12 weeks.

Items	Hi 0	Hi 10	Hi 20	Hi 30
Reads	43,654 ± 18,004	46,206 ± 14,511	37,146 ± 6815	41,233 ± 9479
Observed OTUs	24.3 ± 9.4	24.7 ± 3.9	23.9 ± 6.9	30.0 ± 10.9
Chao 1	27.1 ± 9.9	26.8 ± 5.1	30.6 ± 12.0	36.5 ± 10.6
Shannon_e	1.0 ± 0.4 ^a^	0.7 ± 0.4 ^a^	0.2 ± 0.3 ^b^	0.3 ± 0.3 ^b^
Simpson	0.5 ± 0.2 ^b^	0.6 ± 0.2 ^b^	0.9 ± 0.2 ^a^	0.9 ± 0.2 ^a^
Berger_parker (1/d)	0.6 ± 0.2 ^b^	0.7 ± 0.2 ^b^	0.9 ± 0.1 ^a^	0.9 ± 0.1 ^a^

Reported data are expressed as means ± SD (*n* = 9). The means were compared by ANOVA (*p* < 0.05). ^a,b^ Different superscript letters on the same row indicate significant differences after Tukey–Kramer post-hoc test.

**Table 3:** Results of Permutational multivariate analysis of variance (PERMANOVA) and Analysis of similarity (ANOSIM) based on Bray–Curtis and binary Bray–Curtis dissimilarities using abundance data of mucosa-associated bacterial communities. Significant *p*-values are in bold.

Statistics	Bray–Curtis		Binary Bray–Curtis	
PERMANOVA				
Permutation N	999		999	
Total sum of squares	1.383		0.448	
Within-group sum of squares	0.929		0.274	
F	5.202		6.785	
*p* (same)	**0.001**		0.001	
Pairwise comparisons	*p*-value	F-value	*p*-value	F-value
Hi 10 vs. Hi 0	0.708	2.144	**0.006**	9.156
Hi 20 vs. Hi 0	**0.012**	12.350	**0.012**	9.849
Hi 30 vs. Hi 0	0.078	7.191	**0.006**	12.450
Hi 10 vs. Hi 20	**0.042**	5.824	**0.030**	5.189
Hi 10 vs. Hi 30	0.486	2.691	0.156	2.490
Hi 20 vs. Hi 30	1.000	1.052	0.564	1.934
ANOSIM				
Permutation N	999		999	
R	0.252		0.480	
*p* (same)	**0.002**		**0.001**	
Pairwise comparisons	*p*-value	R	*p*-value	R
Hi 10 vs. Hi 0	0.468	0.133	**0.006**	0.585
Hi 20 vs. Hi 0	**0.018**	0.515	**0.006**	0.703
Hi 30 vs. Hi 0	**0.036**	0.386	**0.006**	0.765
Hi 10 vs. Hi 20	**0.042**	0.344	**0.030**	0.425
Hi 10 vs. Hi 30	0.534	0.130	0.348	0.159
Hi 20 vs. Hi 30	1.000	0.008	0.180	0.190
SIMPER				
Hi 10 vs. Hi 0	26.16		16.77	
Hi 20 vs. Hi 0	32.75		19.23	
Hi 30 vs. Hi 0	31.27		18.33	
Hi 10 vs. Hi 20	25.81		16.09	
Hi 10 vs. Hi 30	24.79		12.98	
Hi 20 vs. Hi 30	19.24		13.85	

**Table 4:** Mean relative abundance (%) ± SD of the most prevalent phyla, orders, classes, families, and genera found in mucosa samples of rainbow trout fed with four experimental diets. Means in the same row with different letters indicate statistical significance between taxonomic groups’ abundances (*p* < 0.05).

Taxa	Hi 0	Hi 10	Hi 20	Hi 30
Phylum				
*Tenericutes*	56.48 ± 21.74 ^b^	77.06 ± 17.44 ^ab^	93.03 ± 10.12 ^a^	91.52 ± 12.68 ^a^
*Proteobacteria*	38.36 ± 18.26 ^a^	21.07 ± 16.85 ^ab^	5.03 ± 7.51 ^b^	7.69 ± 12.82 ^b^
*Firmicutes*	5.05 ± 8.37	1.84 ± 4.00	1.86 ± 4.34	0.49 ± 0.96
Class				
*Mollicutes*	56.48 ± 21.74 ^b^	77.06 ± 17.44 ^ab^	93.03 ± 10.12 ^a^	91.52 ± 12.68 ^a^
*Gammaproteobacteria*	36.20 ± 18.58 ^a^	20.65 ± 16.55 ^ab^	4.98 ± 7.46 ^b^	7.57 ± 12.78 ^b^
*Betaproteobacteria*	5.01 ± 8.39 ^a^	1.82 ± 4.00 ^a^	1.81 ± 4.34 ^b^	0.37 ± 0.78 ^ab^
*Clostridia*	2.15 ± 2.85	0.41 ± 0.39	0.03 ± 0.07	0.10 ± 0.20
Order				
*Mycoplasmatales*	56.48 ± 21.74 ^b^	77.06 ± 17.44 ^ab^	93.03 ± 10.12 ^a^	91.52 ± 12.68 ^a^
*Alteromonadales*	28.69 ± 20.77	15.85 ± 13.38	4.74 ± 7.46	5.32 ± 12.69
*Aeromonadales*	2.30 ± 2.87 ^a^	1.67 ± 3.10 ^ab^	0.12 ± 0.30 ^ab^	0.11 ± 0.30 ^b^
*Neisseriales*	2.15 ± 2.85 ^a^	0.41 ± 0.39 ^a^	0.03 ± 0.07 ^b^	0.10 ± 0.20 ^ab^
Family				
*Mycoplasmataceae*	53.16 ± 20.49 ^b^	77.06 ± 17.44 ^ab^	93.03 ± 10.12 ^a^	91.52 ± 12.68 ^a^
*Shewanellaceae*	28.69 ± 20.77 ^a^	15.85 ± 13.38 ^ab^	4.74 ± 7.46 ^b^	5.62 ± 12.59 ^ab^
*Aeromonadaceae*	2.53 ± 2.77 ^a^	1.67 ± 3.10 ^ab^	0.12 ± 0.30 ^b^	0.01 ± 0.02 ^b^
*Enterobacteriaceae*	4.31 ± 3.67 ^a^	3.13 ± 4.01^ab^	0.13 ± 0.16 ^b^	1.95 ± 5.33 ^ab^
*Neisseriaceae*	1.60 ± 2.45 ^a^	0.22 ± 0.21 ^b^	0.02 ± 0.05 ^b^	0.01 ± 0.02 ^ab^
*Clostridiaceae*	0.31 ± 0.70	0.17 ± 0.42	0.01 ± 0.01	0.02 ± 0.03
*Actinomycetales*	0.11 ± 0.30	0.03 ± 0.02	0.06 ± 0.06	0.23 ± 0.35
*Pseudomonadaceae*	0.16 ± 0.47	0.00 ± 0.01	0.00 ± 0.00	0.00 ± 0.00
*Staphylococcaceae*	0.03 ± 0.07	0.01 ± 0.01	0.03 ± 0.04	0.07 ± 0.12
*Methylobacteriaceae*	0.00 ± 0.01	0.01 ± 0.01	0.02 ± 0.02	0.01 ± 0.01
*Lactobacillaceae*	0.00 ± 0.00	0.00 ± 0.01	0.00 ± 0.01	0.02 ± 0.03
*Enterococcaceae*	0.00 ± 0.00	0.00 ± 0.00	0.00 ± 0.00	0.01 ± 0.02
Genus				
*Mycoplasma*	56.48 ± 21.74 ^b^	77.06 ± 17.44 ^ab^	93.03 ± 10.12 ^a^	87.24 ± 15.81 ^ab^
*Shewanella*	28.69 ± 20.77 ^a^	15.85 ± 13.38 ^ab^	4.74 ± 7.46 ^b^	9.93 ± 16.53 ^ab^
*Aeromonas*	2.30 ± 2.87 ^a^	1.67 ± 3.10 ^ab^	0.12 ± 0.30 ^ab^	0.01 ± 0.02 ^b^
*Citrobacter*	2.83 ± 3.15 ^a^	0.41 ± 0.62 ^ab^	0.04 ± 0.06 ^b^	1.28 ± 3.69 ^ab^
*Kluyvera*	1.72 ± 2.23 ^a^	1.31 ± 2.02 ^ab^	0.01 ± 0.02 ^b^	0.57 ± 1.67 ^ab^
*Yersinia*	0.23 ± 0.58	1.40 ± 3.64	0.07 ± 0.11	0.00 ± 0.01
*Deefgea*	1.66 ± 2.42 ^a^	0.22 ± 0.21 ^a^	0.02 ± 0.05 ^b^	0.01 ± 0.02 ^ab^
*Clostridium_sensu_stricto*	0.28 ± 0.71	0.17 ± 0.42	0.01 ± 0.01	0.02 ± 0.03
*Pantoea*	0.27 ± 0.66	0.00 ± 0.00	0.00 ± 0.00	0.01 ± 0.02
*Pseudomonas*	0.16 ± 0.47	0.00 ± 0.01	0.00 ± 0.00	0.00 ± 0.00
*Staphylococcus*	0.03 ± 0.07	0.01 ± 0.01	0.03 ± 0.04	0.07 ± 0.11
*Methylobacterium*	0.01 ± 0.01	0.01 ± 0.01	0.02 ± 0.02	0.01 ± 0.01
*Lactobacillus*	0.00 ± 0.00	0.00 ± 0.01	0.00 ± 0.01	0.02 ± 0.03
*Enterococcus*	0.00 ± 0.00	0.00 ± 0.00	0.00 ± 0.00	0.01 ± 0.02

^a,b^ Different superscript letters on the same row indicate significant differences after Tukey–Kramer post-hoc test.

## Supplementary Materials

The following are available online at https://www.mdpi.com/2076-2615/9/4/143/s1, Table S1: List of OTUs and corresponding number of reads found in trout intestinal mucosa samples.
